# The high prevalence of *Mycobacterium tuberculosis* Beijing strain at an early age and extra-pulmonary tuberculosis cases

**Published:** 2017-12

**Authors:** Hanifeh Erie, Hami Kaboosi, Naeme Javid, Hesamaddin Shirzad-Aski, Masoumeh Taziki, Maya Babaee Kuchaksaraee, Ezzat Allah Ghaemi

**Affiliations:** 1 Department of Microbiology, Ayatollah Amoli Branch, Islamic Azad University, Amol, Iran; 2 Department of Microbiology, Golestan University of Medical Sciences, Gorgan, Iran; 3 Infectious Diseases Research Center, Golestan University of Medical Sciences, Gorgan, Iran; 4 Tuberculosis Laboratory Center, Golestan University of Medical Sciences, Gorgan, Iran; 5 Laboratory Sciences Research Center, Golestan University of Medical Sciences, Gorgan, Iran

**Keywords:** *Mycobacterium tuberculosis*, Beijing, Pulmonary tuberculosis, Extra-pulmonary tuberculosis, Real-time PCR

## Abstract

**Background and Objectives:**

Tuberculosis (TB) is still responsible for a wide range of deaths worldwide. Beijing genotype is one of the most important and virulent strains in *Mycobacterium tuberculosis*. This study was designed for determination Beijing genotypes of *M. tuberculosis* in Golestan province, north of Iran.

**Materials and Methods:**

In the current descriptive study, 238 clinical MTB isolates, obtained from patients with pulmonary and extra-pulmonary TB in north of Iran, were evaluated. Oligonucleotide primers for the Beijing and non-Beijing genotypes and specific probes for their detection by a real-time PCR method were employed. In addition, an association between the Beijing genotype and possible clinical and demographic factors was evaluated.

**Results:**

The method revealed that 33 cases (13.9%) were the Beijing lineage and 205 (86.1%) the non-Beijing genotype. The mean age of patients infected with the Beijing and non-Beijing strains was 37.27 ± 18.3 and 51 ± 21.2 years, respectively; the difference was statistically significant (P = 0.001). In addition, the prevalence of the Beijing strain decreased with age. Patients with a TB infection caused by the Beijing genotype were also more vulnerable to treatment failure. Based on the origin of the samples, the Beijing genotype was more often observed in extra-pulmonary samples compared with Pulmonary ones (P = 0.001).

**Conclusion:**

The Beijing genotype of MTB is prevalent in our region especially among young people which could indicate the risk of further expansion in the future.

## INTRODUCTION

Despite improvements in global health and medical science, tuberculosis (TB) is still responsible for a wide range of deaths worldwide. *M. tuberculosis* (MTB) is the causative agent of two main types of TB: pulmonary and extra-pulmonary infections (1). Several important genetic lineages including Haarlem, W/Beijing, Latino-American and Mediterranean (LAM), Central Asian (CAS) and East-African-Indian (EAI) were identified in MTB in the past decades (2). Among them, the Beijing strain is responsible for many epidemics of TB. Van Soolingen et al. (1995) were the first to identify the Beijing genotype in China (3). It was reported that the prevalence of this lineage increased in the global population throughout East Asia and the countries of the former Soviet Union; more than half of TB cases can be attributed to this genotype in Asia (2, 4, 5).

Several scientists have reported that the Beijing genotype has some unique properties that make it more virulent than other MTB genotypes (6–10). Moreover, some studies suggested that this strain has a high dissemination power and could spread between different countries (11, 12). In addition, risk factors such as age less than 25 years and co-infection with human immunodeficiency virus (HIV) may be associated with the Beijing strain infection (13, 14). Other studies reported that treatment failure is more common with the Beijing strain (11). Thus, it is important to understand the prevalence of the Beijing strain in specific geographical regions and calculate its spread and growth rate in that area. Furthermore, the relationship between the Beijing strain and factors such as age, gender, treatment failure, and disease type should also be explored.

Rapid and accurate detection methods are the most effective ways to successfully treat and control TB. Differentiation between the strains of MTB is also important to determine the relationship between the strains and the disease (15). Hence, specific diagnostic tools that can rapidly identify MTB cases and simultaneously differentiate between the strains are needed. In recent studies, molecular tools such as IS*6110* DNA fingerprinting, spoligotyping, and real-time PCR have been used to identify different strains of MTB (16–18). The first two methods require expensive equipment and are time-consuming. In contrast, real-time PCR eliminates the limitations of other detection methods and is a sensitive, fast, and accurate method to identify MTB and detect the Beijing strain (15). Hillemann et al. used two specific pairs of primers to detect the Beijing and non-Beijing genotypes using a multiplex real-time PCR assay (18). They successfully detected the Beijing and non-Beijing strains in clinical samples. In the current work, the similar method used to evaluate the prevalence of the Beijing and non-Beijing genotypes in patients with TB admitted to Gorgan Central Hospital in Gorgan, Iran.

Due to the higher global prevalence of pulmonary TB, few studies focused on extra-pulmonary TB (19). As a result, little information exists regarding the impact of various factors on extra-pulmonary TB. Therefore, in the current study, both pulmonary and extra-pulmonary TB infection samples were evaluated.

Currently, the incidence of MTB, particularly the Beijing, is very low in Iran. However, the countries on the Eastern and Northern borders of Iran (e.g., Afghanistan, Pakistan, Azerbaijan, Armenia and the other countries of the former Soviet Union) are the main sources of the Beijing genotype. Due to the high dissemination likelihood of this strain, these countries can transmit the Beijing genotype from Central Asian to Eastern European countries via Iran (20). Therefore, TB prevalence should be updated annually to provide new data about the incidence rate of TB infection caused by the Beijing strain in this region. This study aimed at determining the frequency of the Beijing strain in positive cultures of patients with TB and identifying possible factors affecting its incidence.

## MATERIALS AND METHODS

### Clinical samples, *M. tuberculosis* strains, and patients characteristics.

This descriptive study evaluated 238 clinical MTB isolates obtained from clinical specimens acquired from patients with TB referred to the Mycobacteriology Laboratory at Central Hospital in Gorgan, Iran, from 2010 to 2011. The clinical and demographic information of the patients, including the history of TB, response to treatment, gender, age, and drug susceptibility testing results (if any) were recorded in the Statistical Package for the Social Sciences (SPSS), version 16 (SPSS Inc., Chicago, IL, USA). The Scientific Board of the National Research Institute of Tuberculosis and Lung Disease of Iran reviewed and approved the current study.

Each isolate was sub-cultured on Lowenstein-Jensen (LJ) medium (Oxoid, UK) and incubated at 37°C. Plates were monitored weekly for the growth of bacteria over an 8-week period. Some conventional methods, including colony and growth characteristics, Ziehl-Neelsen staining, pigmentation, nitrate reduction, and production of niacin, were used to verify each colony.

### DNA extraction.

The boiling method was employed to extract DNA according to Reischl et al. (1994) protocol with slight modifications (21). In brief, a loopful of each colony was picked up from LJ culture and homogenized in 100 μL of lysis buffer by vortexing; the lysis buffer contained 1% Triton X-100, 0.5% Tween 20, 10 mM Tris-HCl (pH 8.0), and 1 mM ethylenediaminetetraacetic acid (EDTA). The suspension was incubated at 80°C for 20 min, and then, centrifuged for 1 min at 12000 rpm to sediment the debris. The clear supernatant was transferred to a new microtube and stored at −20°C. The concentration and purity of the extracted DNA were determined using spectrophotometry at 260 and 280 nm wavelengths (Nanodrop ND-1000; Thermo Scientific, MA, USA).

### Real-time PCR.

The real-time PCR assay was conducted by ABI 7300 Real-time PCR system (Foster City, CA, USA) according to Hillman et al. (2006) protocol (18). The technique performed in two separate tubes to identify Beijing and non-Beijing DNA. Oligonucleotide primers and specific probes for the Beijing and non-Beijing genotypes are shown in Table 1. The final volume of each reaction was 25 μL containing 12.5 μL ABI TaqMan Universal PCR Master Mix (Applied Biosystems, USA), 1 μL of each primer, 0.5 μL of the specific probe, 8.5 μL of distilled water, and 1.5 μL of the DNA template. After the initial denaturation at 95°C for 15 minutes, a 40-cycle amplification program was conducted at 92°C for 15 seconds and then, at 60°C for 1 minute.

**Table 1.** Nucleotide sequences used as primers and TaqMan probes for identification of Beijing and non-Beijing strains (18)

**Primers/probe names for target strains**	**Target**	**Sequence**
nBjF	Non-Beijing strains	5′-AAGCATTCCCTTGACAGTCGAA-3′
nBjR		5′-GGCGCATGACTCGAAAGAAG-3′
nBjTM		5′-6FAM-TCCAAGGTCTTTG-MGB-NFQ-3′a
BjF	Beijing strains	5′-CTCGGCAGCTTCCTCGAT-3′
BjR		5′-CGAACTCGAGGCTGCCTACTAC-3′
BjTM		5′-YAK-AACGCCAGAGACCAGCCGCCGGCT-DB-3′b

a MGB-NFQ, minor groove binding, nonfluorescent quencher.

b YAK-DB, Yakima yellow-DABCYL (nonfluorescent dark quencher).

Baseline and threshold values were set to determine the threshold cycle (CT) for the amplification curves. A positive result was defined when a typical sigmoid fluorescence curve was observed for each sample and the final fluorescence intensity was above a CT, which was determined by comparing the fluorescence intensity between the Beijing and/or non-Beijing reference genotypes and the background noise. *M. tuberculosis* H37Rv (the non-Beijing control), a wild type of the Beijing strain (verified by spoligotyping), and a Milli-Q water (Merck Millipore, Germany) were included as controls.

### Data analysis.

The SPSS software was used to analyze the relationship between genotypes of MTB and the studied parameters of participants. Nominal variables were analyzed by the Chi-square test and the Fisher exact test whenever necessary. P ≤ 0.05 was considered statistically significant.

## RESULTS

TB isolates were obtained from 133 (55.9%) male and 105 (44.1%) female samples. Among these 238 participants, 11 (4.6%) were 0–12 years old, 20 (8.4%) were aged 13–20 years, 87 (36.5%) were 21–45 years old, 77 (32.4%) were 46–65 years old, and 43 (18.1%) were 65 years of age or older. The details and analysis results of the samples are shown in Table 2.

**Table 2.** Prevalence of Beijing and non-Beijing genotypes based on Characteristics of samples.

**Variable**	**Genotype**	
	Beijing, N = 33	Non-Beijing, N = 205
**Gender**		
Male, N = 133 (%)^a^	18 (13.5)	115 (86.5)
Female, N = 105 (%)	15 (14.3)	90 (85.7)
**The origin of the samples**		
Pulmonary, N = 219 (%)	26 (11.9)	193 (88.1)
Extra-pulmonary, N = 19 (%)	7 (36.9)	12 (63.1)
**Age (year)**		
1 – 12, N = 11 (%)	4 (36.3)	7 (63.7)
13 – 20, N = 20 (%)	5 (25)	15 (75)
21 – 45, N = 87 (%)	13 (14.9)	74 (85.1)
46 – 65, N = 77(%)	11 (14.3)	66 (85.7)
> 65, N = 43 (%)	0 (0)	43 (100)

a The percentage is calculated based on the number of samples located in horizontal columns.

Out of the 238 MTB samples examined in this study, 33 were the Beijing strain (13.9%), while 205 cases (86.1%) were the non-Beijing. Among the Beijing samples, 15 (45.5%) isolates obtained from females and 18 (54.5%) from males. There was no significant relationship between the Beijing geno-type and the gender of patients. The mean age of the patients infected with the Beijing and non-Beijing strains were 37.27 ± 18.3 and 51 ± 21.2 years, respectively; the difference was statistically significant (P = 0.001). The highest prevalence of the Beijing strain was observed in children under 12 years old (36.4%), and the prevalence of this genotype decreased with age. Furthermore, the Beijing strain was not observed in any patients older than 65 years.

In this study, treatment failure was also analyzed and the results showed that of four cases of treatment failure, three samples were related to the Beijing strain. Based on the origin of the samples, the Beijing genotype was more common in extra-pulmonary samples (36.1%) compared with pulmonary ones (11.9%; P = 0.001).

## DISCUSSION

MTB is an obligate human pathogen that causes TB across the world (1). Due to the high prevalence and dissemination of the disease globally, it is essential to have a rapid and accurate method to detect TB and identify its strains to avoid financial loss (15, 18). Hence, in the present study, the real-time PCR assay was used to evaluate the prevalence of the Beijing and non-Beijing strains in the North of Iran.

In general, the worldwide spread of the MTB Beijing genotype varies from 0 to 80 percent depending on the geographic location. The highest prevalence is observed in the Southeastern countries of Asia (1, 2). In a previous study, among 3634 MTB strains isolated in China, 63% were of the Beijing strain (13). In another investigation, Kam et al. (2005) detected a 68.5% prevalence rate for the Beijing genotype in Hong Kong (22). In total, the average prevalence of the Beijing strain in Iran is estimated to be below 6% (20, 23). As expected, the results of the current investigation revealed a higher prevalence rate for the Beijing strain (13.9%) than its average prevalence in Iran. As previously mentioned, the Beijing genotype is relatively high in the North of Iran. Among 65 MTB isolates in Pakistan, Arif et al. (2014) found 8.9% of them as Beijing strain (24). In another similar study, Niemann et al. (2010) reported a moderate prevalence of the Beijing strain (26%) in Georgia (25). These results emphasized the important role of immigration in the spread of TB. No correlation was observed between the prevalence of the Beijing strain and gender in this study, which was in agreement with the results of previous studies (26).

Results of the present study indicated that the prevalence of the Beijing strain in patients under 12 years old was higher than that of any other age groups. In addition, detection of the Beijing genotype in 1- to 5-year-old children suggests the importance of this genotype. Because children and youth can easily transmit the infection, the increasing incidence of TB caused by the Beijing strain in this region is of great concern. The result is also consistent with the findings of other studies. In two similar studies, Rohani et al., (2009) and Pang et al., (2012) suggested that the mean age of the patients affected by the Beijing genotype was lower than that of the patients infected with other genotypes (13, 27). Pang et al. (2012) proposed that the emergence of the Beijing strain in China goes back less than a century; hence, the elderly are likely colonized with non-Beijing strains, and therefore, they cannot carry the Beijing strain. Consequently, younger people are primarily colonized by the Beijing strain (13). The results of current study represent a clear example of the active transmission of the Beijing strain (among young people) in this region, which could be due to the spread of the strain through the Northern border of Iran. Based on these results, latent infection of young people can be involved in this event.

Of four cases that demonstrated a treatment failure of TB at the end of the second month, three patients were the Beijing-positive. It is noteworthy that a recurrent TB case was found during the study that was also affected by the Beijing strain. Although the number of treatment failure cases was low in this study, the results indicated that the treatment of TB infection caused by the Beijing strain is still difficult. Accordingly, it is vital to identify cases affected by the strain. The increased virulence of Beijing strain might also result in a lower response to treatment. Several cross-sectional studies reported a higher rate of recurrent TB in patients infected with the Beijing strain (13, 28). Lan et al. (2003) found that one of the significant risk factors for treatment failure in TB infection can simply be the infection with the Beijing genotype (28). Similar to the results of this research, Kong et al. (2007) reported that the prevalence of the Beijing strain in patients with extra-pulmonary TB was nearly three times higher than that of the patients with pulmonary TB (19). This difference might be explained by the fact that the Beijing strain can be more virulent and has more capability to invade the extra-pulmonary tissues than any other strains (11). However, further pathogenicity studies are needed to support this theory.

In conclusion, the present study revealed the increased incidence of TB infection caused by the Beijing strain in the North of Iran, compared with previous findings. Furthermore, the Beijing strain may have a higher capacity to resist TB treatment. Hence, to ensure a successful TB control program, certain criteria should be fulfilled, including rapid detection of the disease and its strains, rapid drug susceptibility testing, continuous community-based surveillance, and government monitoring of the border, and increasing the community awareness. Future research should focus on the molecular epidemiology of TB in high-burden areas and their surrounding regions simultaneously, and follow and design programs for controlling the active transmission of TB infection in such areas. Extra-pulmonary TB should also receive more attention in such programs.
